# Supplementary material for: Structural basis of anticancer drug recognition and amino acid transport by LAT1
Source: Nat Commun. 2025 Feb 14;16:1635. doi: 10.1038/s41467-025-56903-w (PMC11828871; doi:10.1038/s41467-025-56903-w)
Supplement: Supplementary file 1 — Supplementary Information [file 41467_2025_56903_MOESM1_ESM.pdf]

Supplementary Information for

Structural basis of anticancer drug recognition and amino acid transport by LAT1

Yongchan Lee, Chunhuan Jin, Ryuichi Ohgaki, Minhui Xu, Satoshi Ogasawara, Rangana Warshamanage, Keitaro Yamashita, Garib Murshudov, Osamu Nureki, Takeshi Murata, Yoshikatsu Kanai

This PDF file contains:

Supplementary Figures 1–8

Supplementary Tables 1–2

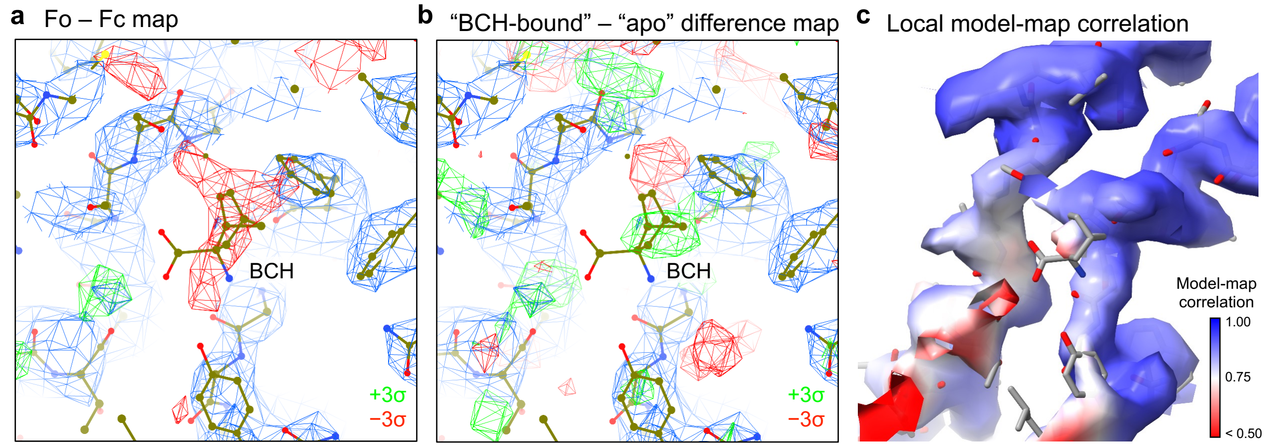

Supplementary Figure 1 | Re-analysis of a previously reported "BCH-bound" structure (PDB ID: 6IRT and EMDB ID: EMD-9722)

a) Fo – Fc map calculated for the "BCH-bound" structure [Yan *et al.*, *Nature*, 2019] using the deposited PDB coordinate (6IRT) and the MRC map (EMD-9722). The blue mesh represents the original unprocessed map, and the green and red meshes represent the positive and negative Fo – Fc densities contoured at +3σ and −3σ (normalized within the mask), respectively. Since the original B-factors were set to 20.00 for all atoms of BCH in the deposited coordinates, the difference map was calculated after 10 cycles of coordinate and ADP refinement. After the refinement, the average B-factor of BCH has increased to 192.7 (ranging from 156.8 to 216.3), well above those of the surrounding atoms (eight residues within 4 Å of BCH), ranging from 25.0 to 115.3. The negative densities overlapping with BCH indicates no binding or low occupancy of the ligand.

b) A difference map between the "BCH-bound" (EMD-9722) and the "apo" maps (EMD-9721). The positive (green) and negative (red) densities were contoured at +3σ and −3σ (normalized within the mask), respectively. There is only a weak positive density partially overlapping with BCH, indicating no binding or low occupancy of the ligand.

c) Local model-map correlation calculated for the "BCH-bound" structure after refinement (6IRT and EMD-9722). The region around the modelled BCH shows poor correlation, probably due to the forced placement of the BCH in a weak density.

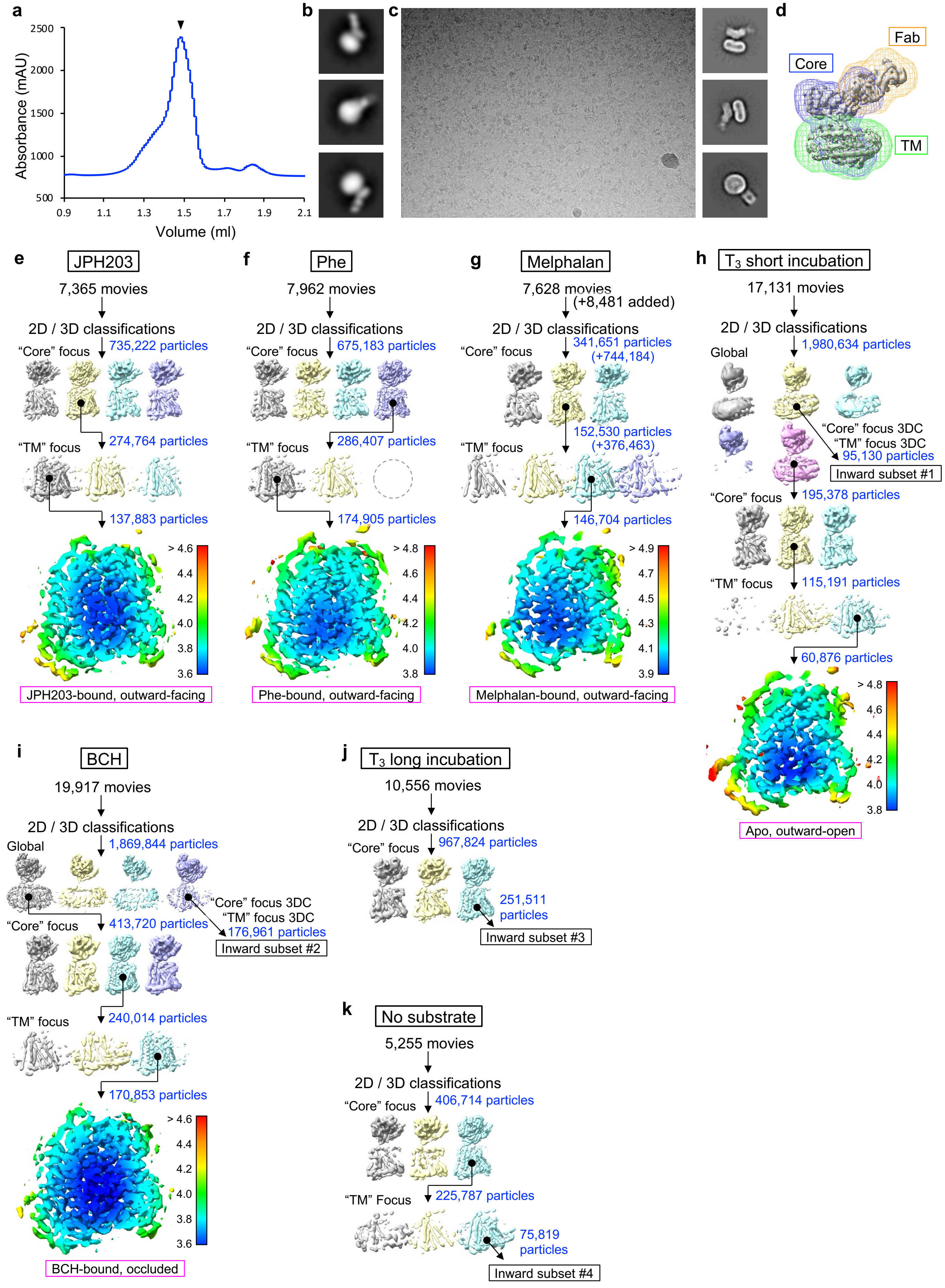

Supplementary Figure 2

Continues on the next page.

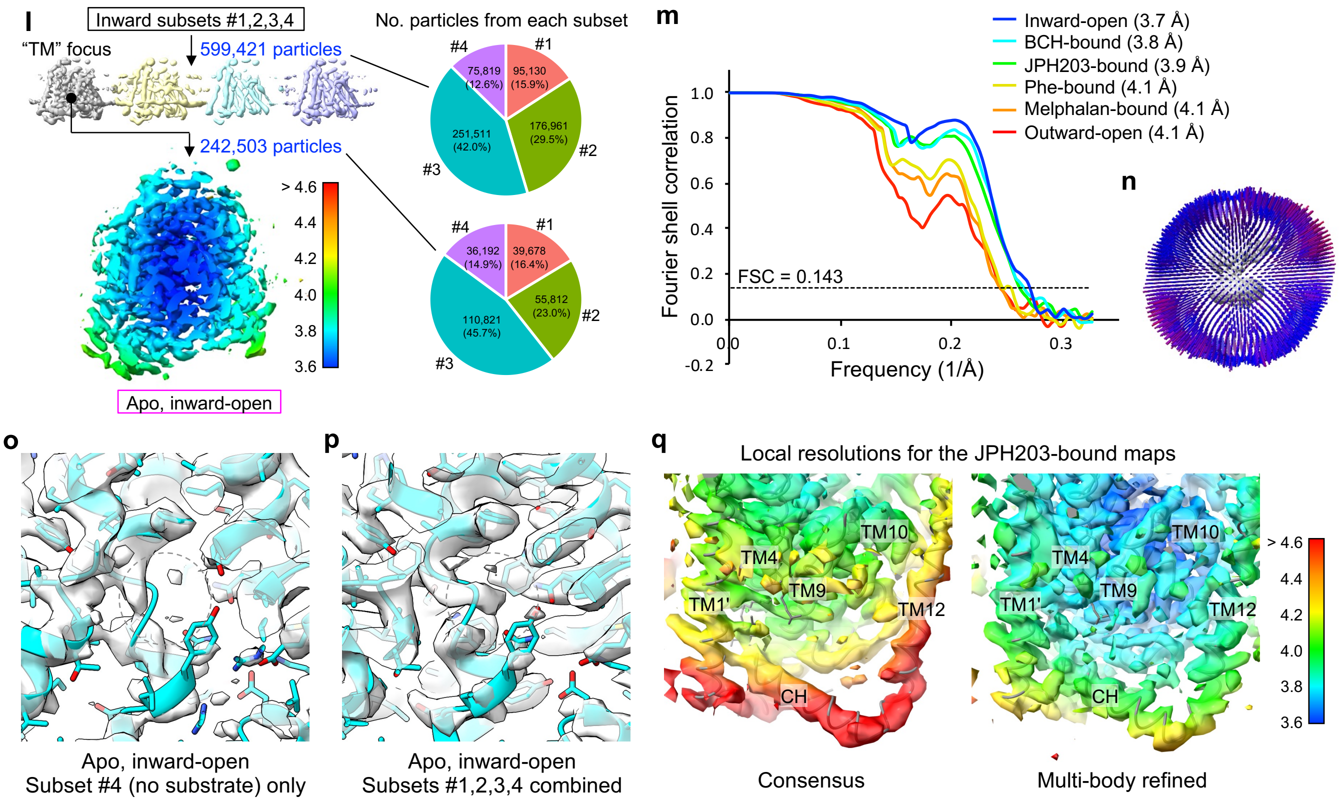

Supplementary Figure 2 | Nanodisc reconstitution, Fab screening and cryo-EM analysis of LAT1–CD98hc

a) Size exclusion chromatography of LAT1–CD98hc in nanodiscs after binding Fab170.

b) LAT1–CD98hc + Fab170 in nanodiscs imaged by negative-stain electron microscopy. Representative 2D class averages are displayed.

c) Representative micrograph and 2D class averages of LAT1–CD98hc + Fab170 bound to JPH203.

d) Masks used for multi-body refinement.

e–k) Single-particle processing workflows of the seven datasets used in this study.

l) Refinement of the apo inward-open structure after combining the particles from four datasets (#1: T_3 short incubation, #2: BCH, #3: T_3 long incubation and #4: no substrate; see also panels **e–k** for details). On the right side, the number and percentage of the particles derived from each subset before and after the classification are plotted as pie charts.

m) Gold-standard half-map FSC curves for the final consensus reconstruction of the six structures. The FSC was calculated based on the phase-randomization procedure as implemented in RELION.

n) The angular distribution of particles used in the final reconstruction of the JPH203-bound data.

o,p) Zoom-up views of the 3D reconstructions of the apo inward-open structure, derived from subset #4 particles only (**n**) or subsets #1,2,3,4 particles combined (**o**). Since the latter showed a better resolution, it was used for all subsequent analyses. Dotted circles indicate the empty substrate-binding site in both maps. See also panels **h–l** for data processing details.

q) Local resolutions for the JPH203-bound maps before and after the multi-body refinement. The identical regions are shown for comparison. The "TM" map from multi-body refinement shows superior map quality and local resolution than the consensus map.

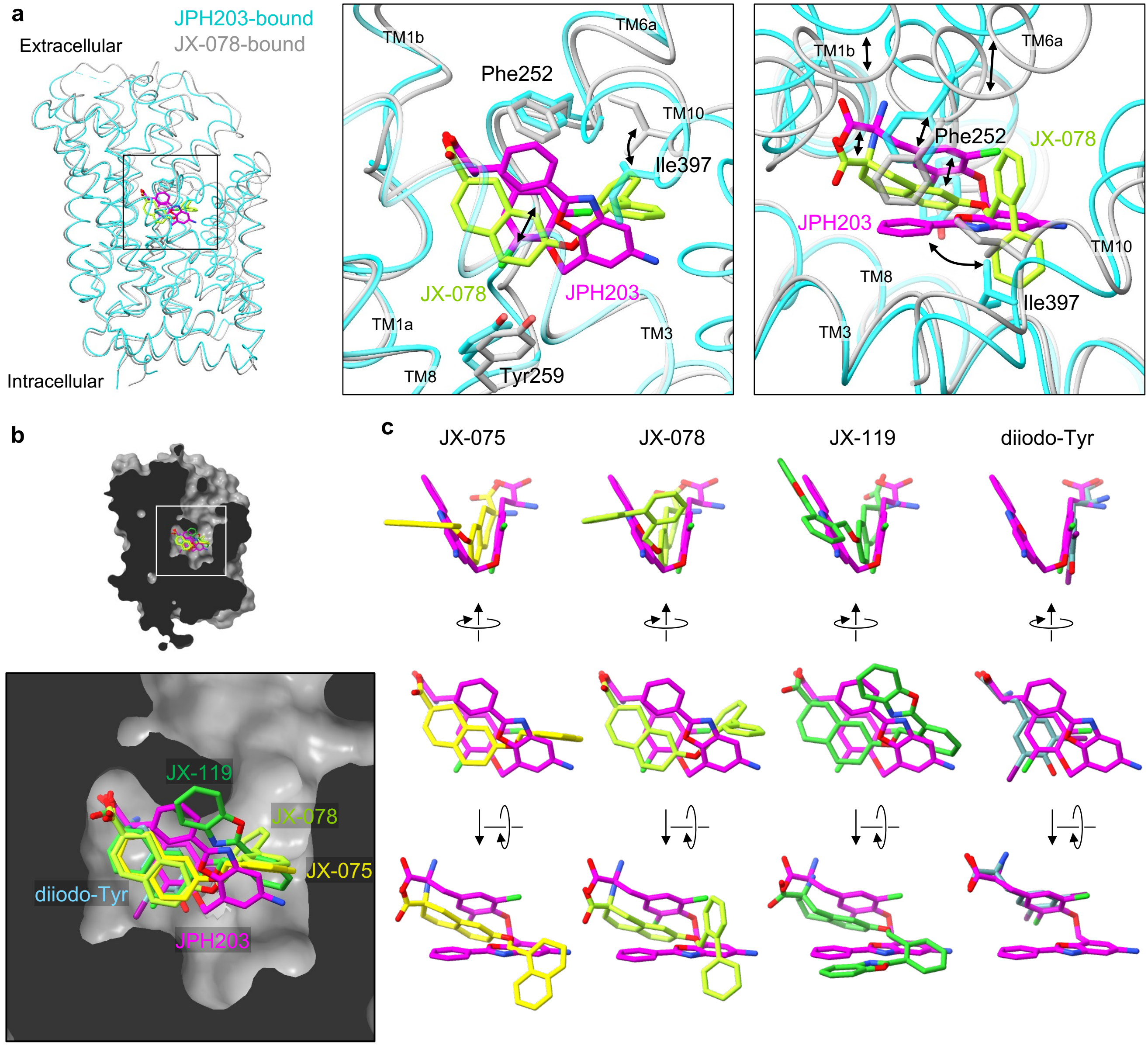

Supplementary Figure 3 | Structural comparison of JPH203 and JX inhibitors

a) Overlay of two LAT1 structures bound to JPH203 or JX-078, superimposed on TM3 and TM8. Zoom-up views highlight the major structural differences of the ligand and the protein depicted as black arrows.

b) Superposition of JPH203, JX-075, JX-078, JX-119 and diiodo-Tyr in the substrate-binding pocket of LAT1. The structures were superposed based onTM3 and TM8. The molecular surface is shown only for the JPH203-bound structure.

c) Overlays of JPH203 to JX-075, JX-078, JX-119 or diiodo-Tyr observed in the pocket of LAT1.

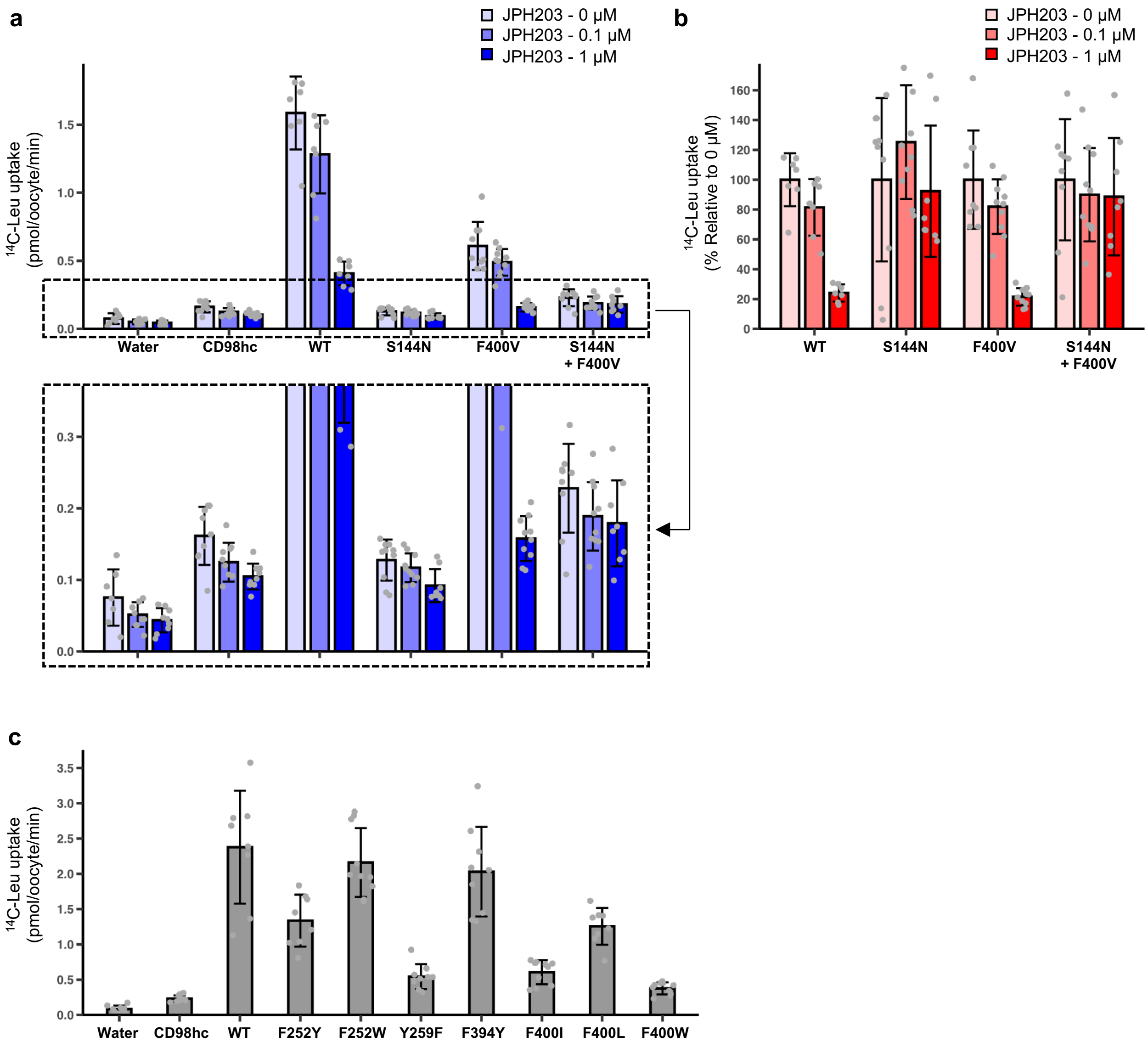

Supplementary Figure 4 | Investigating the activity and the JPH203 selectivity of LAT1 variants

a) The uptake of L-[^{14}C]Leu into *Xenopus* oocytes expressing CD98hc and different variants of LAT1 at increasing concentrations of JPH203 in the external buffer. On the bottom, a zoom-up view is shown to highlight differences of the lower-activity variants. Data are mean ± SD and each data point represents a single oocyte (n = 7–10).

b) The same data as in panel **a**, plotted as % to the reference measured at 0 µM JPH203. The net uptake values were calculated by the subtraction of the negative control (water) at each JPH203 concentration.

c) The activity of the LAT1 variants analyzed. Data are mean ± SD and each data point represents a single oocyte (n = 8–10).

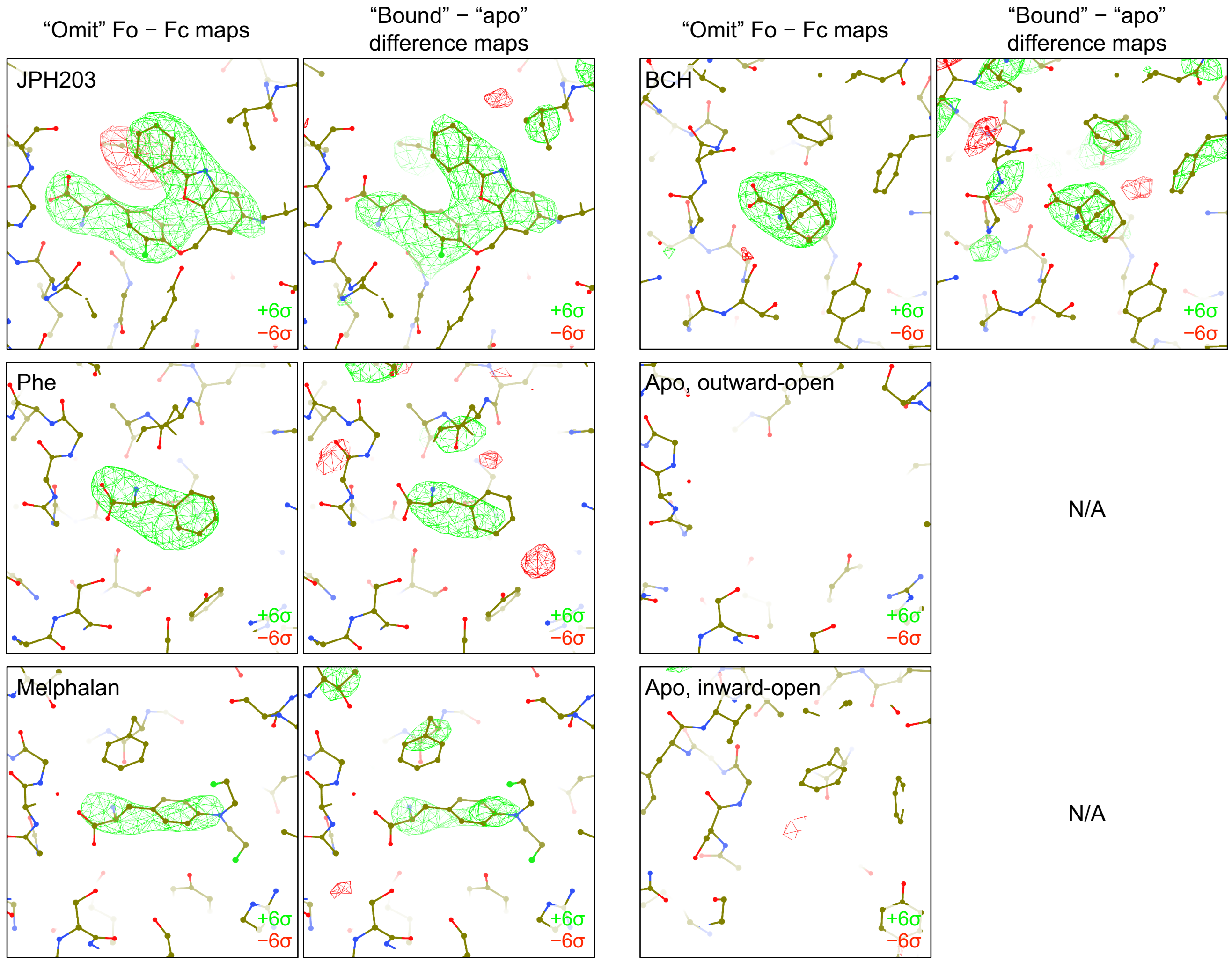

Supplementary Figure 5 | Ligand validation

To validate the presence of ligands, difference maps were calculated using two methods. For the "omit" Fo − Fc density maps (left), the two unfiltered half-maps from RELION and the atomic model without the ligand were used as inputs for Fo − Fc difference map calculation for each ligand-bound data in servalcat. For the apo maps (outward-open and inward-open), Fo − Fc maps were calculated using a similar procedure without a ligand, which showed no significant difference densities. For the Fo − Fo difference maps (right), the ligand-bound maps were first superimposed onto the apo outward-open map using the likelihood-based "overlay" function in EMDA. Difference maps were then calculated for each overlayed pair of maps using the "diffmap" function. Maps have been normalized within the "TM" mask and contoured at +6σ (green) and −6σ (red). Both methods yielded strong positive densities consistent with the expected ligands, confirming the ligand-bound structures. Note that for the BCH-bound structure the difference maps reflect the structural changes between the outward-facing and occluded conformations, showing some positive peaks in the protein region. N/A, not applicable.

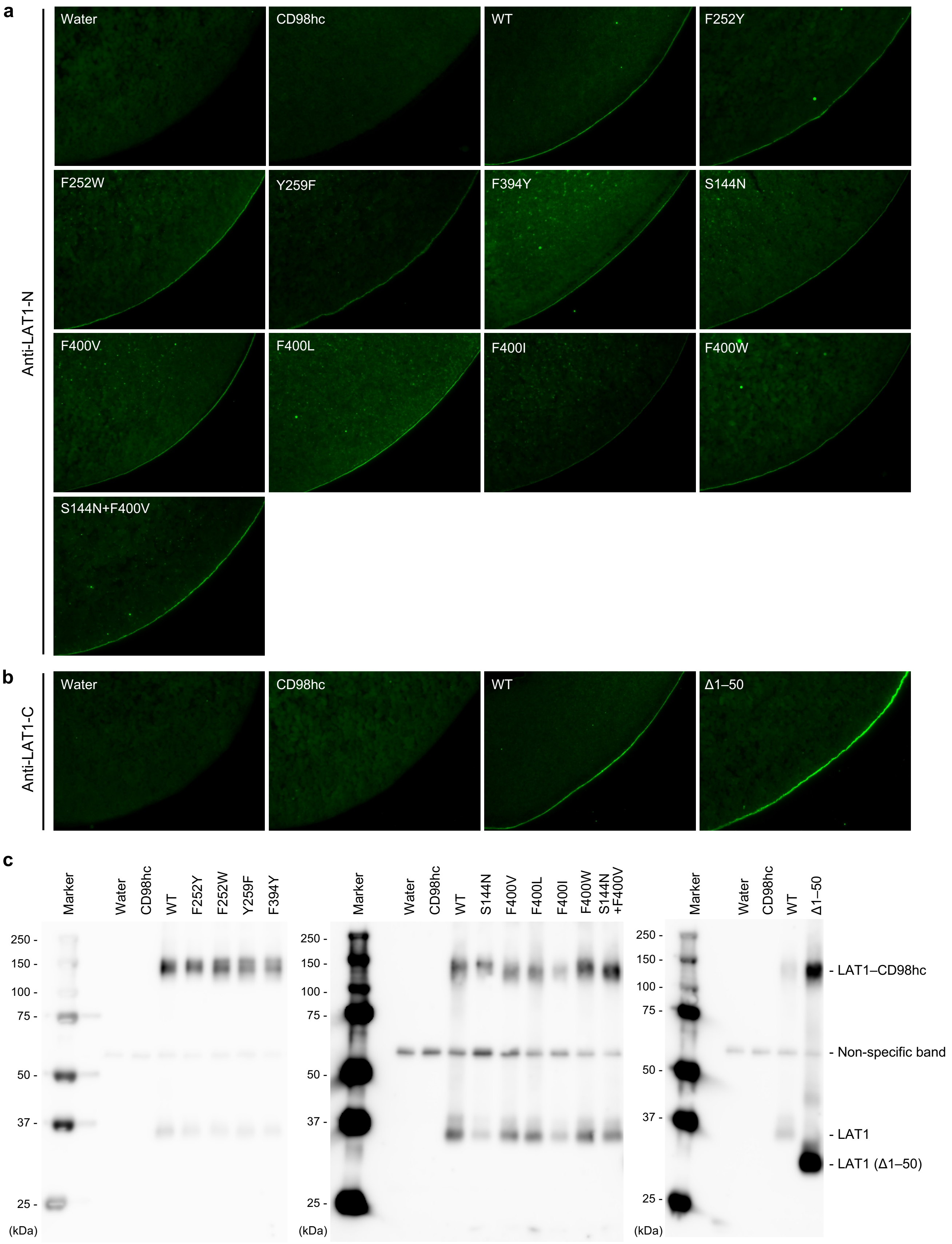

Supplementary Figure 6 | Cell-surface expression of LAT1 mutants in *Xenopus* oocytes

a,b) Immunofluorescence imaging of *Xenopus* oocytes co-expressing CD98hc and different variants of LAT1, using polyclonal antibodies against the N-terminal (**a**) or C-terminal (**b**) region of LAT1.

c) Immunoblot analyses of membrane fractions isolated from *Xenopus* oocytes co-expressing CD98hc and different variants of LAT1, using polyclonal antibodies against the N-terminal region of LAT1.

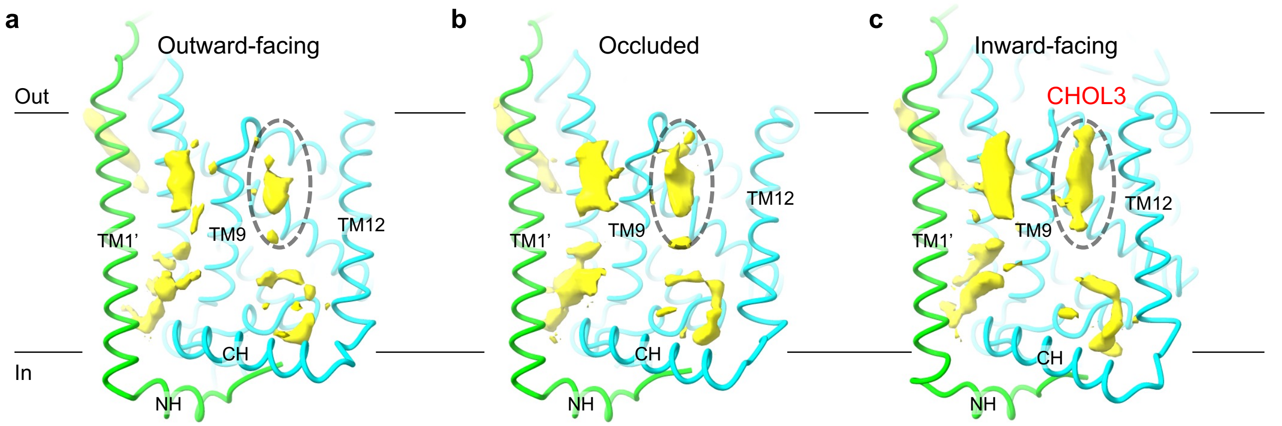

Supplementary Figure 7 | Conformation-specific lipids in LAT1

a–c) Structures of the outward-facing (**a**), occluded (**b**), and inward-facing (**c**) conformations of LAT represented as tube models. All maps showed additional cryo-EM densities around the transmembrane region, most likely representing lipid molecules. One of them, located in a cleft between TM9 and TM12, showed a clear cholesterol-like shape only in the inward-facing conformation (**c**) and becomes blurred when the transporter transitions to the occluded (**b**) and outward-facing (**a**) conformations. The disappearance of the lipid density could be attributed to the dissociation of TM12 away from TM9, which reshapes the protein surface so that cholesterol can no longer bind.

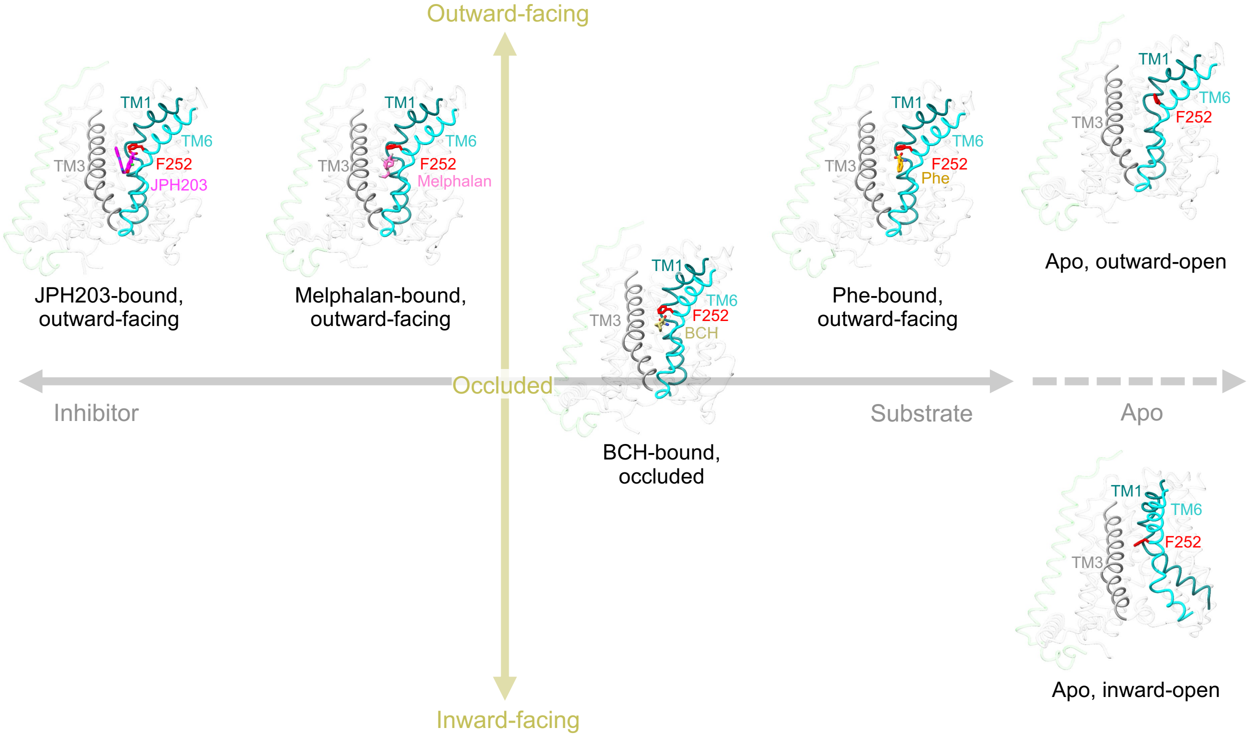

Supplementary Figure 8 | Summary of the experimental structures of LAT1 determined in this study, categorized by conformation and ligand binding state.

Six cryo-EM structures presented in this study are shown as ribbon models and qualitatively categorized by their conformations (vertical axis) and the binding states for inhibitors or substrates (horizontal axis). The apo state is arbitrarily placed at the "substrate" end of the horizontal axis. TM1, TM3 and TM6 are highlighted to illustrate the rocker-switch motion involved in the substrate transport. Phe252 is highlighted as a red stick model to illustrate side chain movement. Ligands are shown as stick models and colored to match the main figures. The extracellular domain of CD98hc has been omitted for clarity.

Supplementary Table 1 | Cryo-EM data collection

	JPH203	Phe	Melphalan	T_3 (short)	BCH	T_3 (long)	No substrate
**Data collection**							
Magnification	105,000	105,000	105,000	105,000	105,000	105,000	105,000
Voltage (kV)	300	300	300	300	300	300	300
Electron exposure (e^{-}/Å^2)	51.0	51.0	51.0	51.0	51.0	51.0	51.0
Defocus range (μm)	−0.8 to −2.0	−0.8 to −2.0	−0.8 to −2.0	−0.8 to −2.0	−0.8 to −2.0	−0.8 to −2.0	−0.8 to −2.0
Calibrated pixel size (Å)	0.837	0.837	0.837	0.837	0.837	0.837	0.837
Initial particles images (no.)[a]	735,222	675,183	1,085,835	1,980,634	1,869,844	967,824	406,714
Particle subsets produced	JPH-bound	Phe-bound	Melphalan-bound	Outward-open<br>Inward-open #1	BCH-bound<br>Inward-open #2	Inward-open #3	Inward-open #4

[a] After particle pre-cleaning with 2D/3D classifications

Supplementary Table 2 | Data processing, model building and validation statistics

	JPH-bound outward-facing		Phe-bound outward-facing		Melphalan-bound outward-facing		Apo outward-open		BCH-bound occluded		Apo inward-open	
**Data processing**												
Particle subsets used	JPH-bound		Phe-bound		Melphalan-bound		Outward-open		BCH-bound		Inward-open #1<br>Inward-open #2<br>Inward-open #3<br>Inward-open #4	
Final particle images (no.)	137,883		174,905		146,704		60,876		170,853		242,503	
Final pixel size (Å)	1.5345		1.5345		1.5345		1.5345		1.5345		1.5345	
Symmetry imposed	C1		C1		C1		C1		C1		C1	
	Consensus	TMD	Consensus	TMD	Consensus	TMD	Consensus	TMD	Consensus	TMD	Consensus	TMD
EMDB ID	EMD-37132	EMD-37134	-	EMD-37140	-	EMD-37141	-	EMD-37142	EMD-37135	EMD-37136	EMD-37137	EMD-37138
PDB ID	8KDD	8KDF	-	8KDN	-	8KDO	-	8KDP	8KDG	8KDH	8KDI	8KDJ
Map resolution (Å)												
Half map FSC = 0.143	3.83	3.89	3.94	4.12	3.89	4.12	3.89	4.12	3.68	3.78	3.58	3.73
Map sharpening *B* factor (Å^2)	–101.5	–138.7	–125.6	–196.8	–133.2	–184.6	–82.2	–145.9	–108.4	–163.0	–98.4	–171.7
Local resolution range (Å)	3.5–5.0	3.6–4.6	3.6–5.4	3.8–4.8	3.6–5.6	3.9–4.9	3.5–6.0	3.8–4.8	3.4–5.0	3.6–4.6	3.2–5.0	3.6–4.6
**Refinement**												
Initial model (PDB codes)	6IRS	6IRS		6IRS		6IRS		6IRS	6IRS	6IRS	6IRS	6IRS
Refinement resolution (Å)	3.6	3.7		3.9		4.0		4.0	3.6	3.7	3.6	3.7
Map-model FSC = 0.5 (Å)	3.6	3.6		3.9		3.9		3.9	3.5	3.7	3.5	3.7
Model composition												
Non-hydrogen atoms	10,626	4,045		3,930		3,937		3,918	10,601	4,022	10,532	4,013
Protein residues	1,361	507		506		506		506	1,361	506	1,353	499
No. ligands	GlcNAc: 4<br>JPH203: 1	CHOL: 2<br>POPC: 1<br>JPH203: 1		Phe: 1		Melphalan<br>: 1		-	GlcNAc: 4<br>BCH: 1	CHOL: 2<br>POPC: 1<br>BCH: 1	GlcNAc: 4	CHOL: 3<br>POPC: 2
Average *B* factors (Å^2)												
Protein	214.2	179.7		237.21		235.1		213.7	213.7	193.5	210.2	216.2
Other	312.8	215.9		204.58		307.7		-	369.2	227.4	369.4	252.9
R.m.s. deviation												
Bond lengths (Å)	0.011	0.011		0.010		0.010		0.009	0.011	0.009	0.012	0.010
Bond angles (°)	1.98	1.89		1.89		1.86		1.86	2.05	1.87	2.03	1.92
**Validation**												
MolProbity score	1.27	1.06		0.93		0.79		0.81	1.51	1.08	1.39	1.13
Clashscore	1.51	1.47		0.62		0.12		0.37	1.89	1.23	1.80	1.11
Rotamer outliers (%)	1.20	0.00		1.17		0.47		0.23	2.07	0.23	1.47	0.48
Cβ outliers (%)	0.79	0.00		0.21		0.21		0.00	0.87	0.43	0.72	0.22
CaBLAM outliers (%)	1.50	0.40		1.20		1.20		1.80	1.40	0.60	1.80	1.60
Ramachandran plot												
Favored (%)	95.41	97.01		97.00		96.40		97.00	95.41	96.40	95.38	95.54
Allowed (%)	4.52	2.99		3.00		3.60		3.00	4.52	3.40	4.54	4.46
Outliers (%)	0.07	0.00		0.00		0.00		0.00	0.07	0.20	0.07	0.00
